# From speech acts to communicative acts: social network debates about sexual consent

**DOI:** 10.3389/fsoc.2024.1468173

**Published:** 2025-01-07

**Authors:** Elisabeth Torras-Gómez, Arja Krauchenberg, Victor Petuya, Rebeca Marcos, Olga Serradell, Marta Soler-Gallart

**Affiliations:** ^1^Vicerectorat de Recerca, University of Barcelona, Barcelona, Spain; ^2^Department of Sociology, Autonomous University of Barcelona, Cerdanyola del Vallès, Spain; ^3^European Parents’ Association, Brussels, Belgium; ^4^Department of Mechanical Engineering, University of the Basque Country, Bilbao, Spain; ^5^University of Barcelona, Barcelona, Spain; ^6^Department of Sociology, University of Barcelona, Barcelona, Spain

**Keywords:** dialogic communicative acts, power communicative acts, consent, social media analytics, gender violence, sociology

## Abstract

**Introduction:**

Understanding consent is essential to combat sexual violence, a deeply rooted social problem. Amidst its complexities, the scientific literature has emphasized the shortcomings of only considering the speech act—whether the victim-survivor said “yes” or not. Instead, sociological research underscores the need to analyze the whole communicative act where different elements lead to either a power relationship where there is no consent or a dialogic relationship where freedom is granted. Although some research has been conducted on citizens’ social media debates on consent, how such debates include the concept of communicative acts to discuss it has not been analyzed yet

**Methods:**

55 gender-related Instagram and Twitter (now known as X) posts—published and extracted over the course of 14 days—were analyzed.

**Results:**

Findings reveal that most posts refer to Power Communicative Acts as a hindrance for consent due to hierarchical power imbalances or to coercion, and called for the need to establish elements of Dialogic Communicative Acts to achieve consent and construct more egalitarian environments. Finally, most posts that considered ethics spoke about the need for perpetrators to be held accountable or offered similar takes on consequentialism.

**Discussion:**

These findings help illustrate how several social media debates about consent successfully fall into the Communicative Acts framework.

## 1 Introduction

Despite recent social movements which have significantly contributed to the conversation on gender based and sexual violence (Lankford, 2016), instances of abuse and harassment continue. The UN reports 35% of women worldwide to have experienced sexual or intimate-partner violence in 2021 (Facts and Figures: Ending Violence Against Women, 2024). Furthermore, sexual violence not only affects women and is not always perpetrated by men. Although to a lesser extent, there are also male victims of sexual harassment, both perpetrated by men and by women (Thomas and Kopel, 2023), and there is also increasing attention paid to the sexual violence suffered by LGBTI+ individuals (Ison, 2019).

Within this worldwide social problem, consent is a widely debated concept across different sciences and citizen debates (Agu et al., 2022) and, as a result, efforts to promote a clear definition of consent have come a long way. Contributions from Psychology, Linguistics, Women Studies or Criminology, among others, have informed legal as well as popularly embraced definitions of consent (Flecha et al., 2020). Furthermore, sociology has made key contributions to better understanding and identifying consent, advancing toward dialogic relationships based on freedom and lack of violence (Flecha, 2022). Similarly, debates throughout social networks reflect a deepening of these conversations about the complexities of consent (Baldwin-White and Gower, 2023; Ison, 2019; Rios-Gonzalez et al., 2024). Social networks have provided a platform for important social movements and activism (Poell, 2014; Zheng, 2020)—namely the #metoo movement—that have contributed greatly to the collective grappling with issues of gender-based violence, such as sexual harassment at the workplace. However, the scientific literature has not yet identified whether and how these wider non-academic conversations fit into the consent debate from the communicative acts framework. This study aims at analyzing the ways in which such debates include the concept of communicative acts to discuss what does and does not constitute consent. This article starts by touching on how the move from speech act theory to communicative acts theory enables a more robust understanding of consent, discussing the importance of distinguishing between Dialogic and Power Communicative Acts to better understand when a relationship is consented or coerced. Next, existing research analyzing social network debates on consent is highlighted. Finally, the analysis of 55 Instagram and Twitter posts through Social Media Analytics (SMA) methodology is introduced, followed by a discussion on the results and concluding remarks.

### 1.1 Scientific contributions, from speech acts to communicative acts, to understanding consent

Within pragmatic linguistics, John Langshaw Austin’s (1962) Speech act theory is considered a founding block of pragmatics and is centered around the distinction between locutions (literal meaning in or of an utterance, e.g., “I like the way you prepare coffee”), illocutions (what one is trying to do through the utterance, e.g., getting someone to make them a cup of coffee) and perlocutions (the effect the utterance has on the world, whether intended by the speaker or not) (Soler and Flecha, 2010). When moving this analysis over to the issue of sexual violence one must identify whether refusal of consent is illocuted. If so, when the perlocutionary act does not respect this refusal and results in a sexual act instead, it is considered a violation (Cowart, 2004). Speech act literature is mainly centered on the first step of this analysis: defining what makes an illocution—e.g. the expression of consent—successful. This set up favors affirmative consent (“no means no” and “only yes means yes”) (Curtis and Burnett, 2017), as it clarifies the expression thereof and makes the distinction between perlocutions that violate and those that misunderstand the victim-survivor’s wishes, easy to differentiate.

Advancements in the communication and conceptualization of consent, including in legislation, have led to define it as affirmative, free and voluntary, moving toward an affirmative model of consent (North, 2022). There is also legislation that considers consent to be informed, mutual, and considered under the person’s full capacity (Vidu Afloarei and Tomás Martínez, 2019). However, terms such as “affirmative,” “voluntary” or “conscious” have meanings full of content and, while they should not be taken for granted, focusing them on the victims’ words poses limitations to fully understand and identify whether a victim truly consented or not. Indeed, victim-survivors often concede to sexual relationships that they do not actually want to engage in for a range of reasons. Many do not consider withdrawal of consent a possibility (Benoit and Ronis, 2022). The self-perceived contradiction between saying “yes” and meaning “no” or saying “yes” at first and “no” further down the line would constitute an ambiguous speech act. Conceptually, however, if an ambiguous speech act is followed by an undesired perlocution—namely sexual violence—this falls under the analytical lens of miscommunication (Cowart, 2004). Thus, it facilitates victim-survivor-blaming attitudes, given that the analysis is centered on the expression of consent, not the abuse itself. The intentionalist take disambiguates these illocutions by simply centering the interpretation of “intended meaning” through the person who gave or refused consent. Yet while this may theoretically be possible it has some practical restrictions. Namely, in a court of law it would be hard to distinguish the victim-survivor’s proclaimed intentions with hearsay (Goldberg, 2020). Furthermore, if intentions are conveyed but do not necessarily influence the perlocutionary outcome, then this theory provides little insight into how consent can be promoted. The consideration of merely affirmative consent has led to the demand for a revision of this concept as it does not respond to the reality experienced in situations where the issue of whether or not there is consent is at stake (Curtis and Burnett, 2017; Willis and Jozkowski, 2018).

While intentionalists point toward the importance of empathy in communication, they still emphasize the need to keep illocutions and perlocutions conceptually separate. As a result, speech act theory cannot account for how understanding is created. Instead, it sets up a conceptual minefield for the illustration of more complex expressions of consent or consent refusal. Ultimately, beyond the support of affirmative consent, speech act theory’s contributions provide limited practical help for defining consent, and the separation of illocutions and perlocutions therefore becomes irrelevant (Dietz and Widdershoven, 1991).

Sociology has provided key concepts and research evidence to overcome such limitations and advance toward a more holistic conceptualization of consent, making relevant contributions to research on gender and sexual violence (Gronert, 2019). The theory of dialogic society (Flecha, 2022; Flecha et al., 2022) has co-created with diverse people (especially oher women who have often been marginalized from mainstream feminist movements) key analyses, theories and empirical evidence that are advancing toward more feminist and egalitarian dialogic societies. Within the dialogic society, sociologists Soler and Flecha co-created the theory of communicative acts (Flecha et al., 2020; Soler and Flecha, 2010). The complexity of how language constructs reality cannot be accounted for by only analyzing spoken language. Other non-verbal elements such as body language, tone, the speaker’s intentions, the context, or the consequences of the communication are necessary to better understand, as in the case of the present study, whether a sexual relationship is based on consent or the lack thereof. Contributing to this distinction, Flecha and Soler develop the concepts of dialogic communicative acts and power communicative acts (Soler and Flecha, 2010). Dialogic communicative acts are those where all parties enjoy equal standing; in which individual freedom is respected and mutual understanding is proactively pursued through honest and non-coerced communication. Power communicative acts are those where a perpetrator will employ whatever means necessary to force or coerce their victim-survivor into doing what they want them to do.

Often perpetrators execute a power communicative act by taking advantage of pre-existing power asymmetries or by imposing themselves on the victim-survivor otherwise. Flecha et al. (2020) thus outline two types of power that are available to abusers and rapists to engage in power communicative acts. On the one hand, institutional power represents a social structure that has real economic and other social implications for all members of those institutions, e.g., the workplace. Moreover, institutions tend to have organizational hierarchies which grant some members the power over said repercussions. Thus, a victim-survivor may feel forced to engage in certain sexual acts in order to avoid being fired from their job, particularly if the perpetrator is a superior to the victim-survivor. On the other hand, Interactive power refers to the power provided by the interactions people engage in, which might include coercion, insisting, or being manipulative, among others (Racionero-Plaza et al., 2022). The imposition of power of one person over another occurs by the interaction itself, determined by the context. A person may threaten to spread lies or use any other manipulative and coercive tactic to intimidate the victim-survivor into giving in.

Abusers often take advantage of institutional or interactive power without explicitly mentioning it and yet—as pointed out earlier—are often aware of their victim-survivor’s silent discomfort. Even in those cases in which the person who holds more power over the other never intended for the power asymmetry to influence the victim-survivor’s decision, with power comes responsibility. Therefore, Flecha et al.’s (2020) theory of communicative acts includes an emphasis on responsibility. Dialogic communicative acts can only happen when abuse is conceptualized with a Weberian (Weber, 1930) understanding of responsibility, in which consequences, not intentions, determine somebody’s culpability (Flecha et al., 2020). Thus, their definition of communicative acts facilitates the integration of a clear understanding of consent which delimitates violent from non-violent sexual encounters, without oversimplifying it through an affirmative “yes” and “no.” The concepts of dialogic vs. power communicative acts seeks to reconcile the complexity of language with the simple distinction of consent vs. no consent by incorporating responsibility and power asymmetries into the analysis of communication (Flecha et al., 2020). Finally, communicative acts analysis shows that institutional and interactive power asymmetries can also be overcome through the commitment to dialogic communicative acts by institutions and by all parties involved.

### 1.2 Consent in social networks

While academia and social media discourse are mostly two separate spheres, the two interact given the medium’s predisposition as a global platform for sharing ideas. Indeed, as more and more citizens turn to social networks to share, express and debate issues of concern, researchers are increasingly analyzing such debates in an attempt to further collect citizens’ voices (Pulido Rodríguez et al., 2020). For instance, analyzing health-related citizens’ debates on social media has been essential to better understand issues of utmost relevance to the public health, such as how misinformation is spreading (Pulido Rodríguez et al., 2020) or vaccine hesitancy (Dredze et al., 2016).

Although to a lesser extent, social network interactions on issues related to consent and violence against women are also being studied (Kettrey et al., 2021; Molnar and Hendry, 2022; Pulido et al., 2023). Especially since #MeToo has gained international resonance across different social networks, a few studies have analyzed the ways in which consent is defined, exemplified, and discussed in different social networks (Aurrekoetxea-Casaus, 2020; Kettrey et al., 2021; Worthington, 2020). For instance, Aurrekoetxea-Casaus (2020) analyzed discussions around consent and sexual violence on Twitter (now known as X) following the publishment of the court’s verdict over the San Fermines Wolf Pack case—in which a group of five men raped a girl during the festivals. Among the tweets analyzed, to what extent the victim-survivor gave consent was discussed as one of the focal points to provide support to the victim-survivor (Aurrekoetxea-Casaus, 2020). Some of these tweets questioned whether the victim-survivor holds responsibility to seek consent, whereas others discussed the lack of conditions the context provided for the victim-survivor to give consent.

However, research on social media discourses on consent does not analyze the ways in which the scientific debates and advancements on consent are taken up and used—or not—by citizens in social network debates. This article aims to integrate the scientific literature on communicative acts with citizens’ social media interactions around consent. In particular, it seeks to better understand to what extent the concept of communicative acts is captured in citizens’ debates on consent—or lack thereof—on Twitter and Instagram.

## 2 Materials and methods

All the authors of this manuscript are sensitized to issues of sexual violence because some of them have suffered Isolating Gender Violence (Flecha et al., 2024) because they have defended, both through research and through personal positioning, victim-survivors of gender violence.

The present study utilizes Social Media Analytics (SMA) methodology (Pulido Rodriguez et al., 2021), which builds on the communicative methodology. The communicative methodology approach engages individuals’ and communities’ voices throughout the entire research in the process of co-creation of evidence and knowledge that will contribute to transforming citizens’ lives, thus achieving social impacts in line with the goals agreed upon by citizens. This methodology has led to the European Commission’s inclusion of co-creation and social impact as a requirement for all research projects funded under Horizon Europe. Within this framework, SMA allows researchers to collect and analyze wide and diverse citizen debates on issues which are of utmost concern to them (Pulido et al., 2023), thus being the optimal methodology to collect citizens’ voices and debates on consent. Following the dialogic basis of the communicative approach, the entire process of the SMA is conducted in an egalitarian dialogue among researchers in order to arrive at consensus based on arguments, or validity claims, rather than on power claims (Pulido Rodriguez et al., 2021).

The bottom-up nature of SMA ensures that scientific concepts are in touch with everyday issues in people’s lives, thus preventing oversimplification and ensuring that the concepts provided are accurate and useful for widespread use. For instance, research using this methodology has shown that social media users interact more with scientific evidence related to COVID-19 than with the fake news that emerged during this pandemic (Pulido Rodríguez et al., 2020). Thus, SMA has the ability to keep the lens fixed on what society demands and needs.

### 2.1 Data collection

The extraction of the data for this study was accessed through a related SMA study executed by ALLINTERACT, a project funded by the European Union’s Horizon 2020 Research Framework Program. The project seeks to generate greater understanding of how societal actors respond to and interact with scientific research in a bid to improve the cooperation between science and society, as well as promoting the engagement of young and vulnerable people in scientific research.

As part of the SMA, a vast extraction of social media posts was conducted across four different platforms by researchers from ALLINTERACT’s leading partner, the University of Barcelona—of which Marta Soler was the KMC Coordinator, Elisabeth Torras-Gómez an official team member, and Rebeca Marcos was hired as a research assistant to provide support in SMA-related tasks. Such platforms were Twitter, Instagram, Facebook and Reddit. To this end, the project selected popular hashtags in each platform related to both gender and education respectively, as well as 10 gender and education related trending topics selected from a pool of the Top 50 Trending Topics on Twitter in all EU countries. Researchers extracted up to the first 10,000 social media posts of each topic and hashtags posted between the 4th and 17th of March 2021.

The present study has utilized this large database and accessed all gender related posts from Twitter and Instagram in it. In total, 54,248 posts from 15 different hashtags were available for analysis. Ten hashtag extractions stemmed from twitter (#EnoughIsEnough; #Equality; #EqualPayDay; #Gender; #GenderEquality; #HeForShe; #IStandWithLinda; #ReclaimTheStreets; #WiMINConference21; #WomenRights) and 5 from Instagram (#DomesticViolence; #Feminism; #GenderEquality; #WomenEmpowerment; #WomenRights). To comb through this extensive dataset and limit the analysis to the scope of the present study, the following keywords related to the topic of consent were searched for: Abuse; Approval; Assault; Awareness; Awkward; Body; Challenge; Choice; Clothes; Coercion; Consent; Dress; Force; Freedom; Gender; Harass*; Kleid; Male Privilege; No Means No; Power; Push; “R*pe”; Rape; Relation; Report; Scared; Security; Sex; Skirt; Smile; Text; “Tw:”; Uncomfortable; Undermine; Victim; Violence; Workplace. Some of these words were selected due to their conceptual relationship to the topic; others were identified in the relevant posts themselves and added to the search thereafter. Not all hashtags were searched for all keywords, but rather we chose from these words inductively depending on the subject matter that was common amongst each hashtag. Table 1 shows the number of posts found, reviewed and analyzed per hashtag in each platform, further explored in the next subsection.

**Table 1:** Posts extracted and analysed in each platform.

Twitter	Total	Reviewed	Analysed
#EnoughIsEnough	10,000	1,454	6
#Equality	10,000	939	1
#EqualPayDay	5,231	27	0
#Gender	9,107	466	2
#GenderEquality	3,514	783	3
#HeForShe	2,487	203	1
#IStandWithLinda	945	60	2
#ReclaimTheStreets	6,803	756	4
#WiMINConference21	186	1	0
#WomenRights	975	108	0
10	49,248	4,797	19

Instagram	Total	Reviewed	Analysed
DomesticViolence	1,000	617	5
Feminism	1,000	330	16
GenderEquality	1,000	229	6
WomenEmpowerment	1,000	96	0
WomenRights	1,000	285	9
5	5,000	1,557	36
Total	54,248	6,354	55

### 2.2 Data analysis

#### 2.2.1 Dialogic codebook: analytical categories

Within the SMA methodology, this study conducted the analysis following the Communicative Content Analysis (CCA), a novel approach to content analysis of social media that seeks to complement evidence-based academic knowledge and its theoretical advancements with the plurality of voices of societal actors and the examples they provide (Pulido Rodriguez et al., 2021). This methodology is based on a Dialogic approach which aids the scientific co-creation of knowledge. In the case of the current research, the process of analysis observes the overlaps between the scientific understanding of the significance of communicative acts for consent on the one hand, and the presence of this concept within popular statements on social media on the other. Once the overlaps are identified, the careful dialogic analysis of each post and its content informs further questions and criteria to include in the study.

Through the keyword search, 6,354 posts were reviewed in an excel sheet to determine whether their content was related to the topic of consent or not for the further analysis of those that were. The unit of analysis of a post included all the content within and related to such post, including all links, pictures and files attached to it as well as the written text contained in it. Those 6,354 posts were analyzed based on the following criteria of inclusion. (1) Posts had to refer to consent, either directly or indirectly. (2) The content had to reflect an understanding of consent as a choice, and thus by definition as something freely given and requiring an egalitarian context. Therefore, posts that merely mentioned the need for consent without pointing at what is included in or excluded from this term were not selected as relevant. (3) posts that referred to the definition of consent but applied an essentialist logic and relied on broad, inaccurate and sexist generalizations, such as “men are responsible for the establishment of consent” were excluded. While we understand that these posts may in some instances be part of a broader, more nuanced conversation, these tweets in isolation did not provide enough information to be included in this study. If all these criteria were met, the tweet or Instagram post was added to a separate file, including the links and the engagement these posts generated (i.e., number of retweets, likes, and comments). If these criteria were not met, the post was excluded. In all, 46 of the reviewed tweets met the aforementioned criteria, meaning they were identified as related to the study’s topic and added to a separate file for the qualitative and quantitative analysis.

Once the 46 posts related to the goal of the present study were found, a dialogic codebook was developed among researchers to further analyze and code the posts. As its name indicates, the codebook was developed dialogically among researchers in order to arrive at a consensus on which categories and subcategories to define for the analysis. Three main categories were established based on the scientific literature on communicative acts theory and on consent: Power Communicative Acts, Dialogic Communicative Acts, and Ethics. Each of them was further broken down into specific subcategories.

Within Power Communicative Acts, two subcategories were defined: Institutional Power and Interactive Power. Interactive Power was further broken down into (1) sexual scripts, referring to the role of shared beliefs and interpretations of sexual behaviors on determining consent or lack thereof; (2) nonverbal cues, understood as nonverbal cues indicating lack of consent, (3) coercion, defined as situations of power with respect to the other in a given context, and (4) unwanted sexual consent, describing situations in which sexual consent is given due to interactions of power, rather than freely given.

The Dialogic Communicative Acts category was divided into (1) nonverbal cues, referring to paying attention to the presence of nonverbal cues indicating consent or lack of consent, and (2) consent support, indicating that making consent visible is appreciated.

Last, the Ethics category was broken down into two categories: (1) intention, concerned with perpetrators’ intention to harass or abuse, and (2) consequences, concerned with perpetrators being held accountable and/or taking accountability for the consequences of their actions. Table 2 describes the codebook.

**Table 2:** Dialogic codebook.

Categories	Subcategories	Definitions
Power communicative acts	Institutional power: Coercion	Talks about one of the persons being in an institutional or hierarchical position of power with respect to the other, who may suffer consequences if he or she refuses to consent (e.g., is his or her superior at work, is a teacher of a student…).
	Interactive power: Sexual scripts	Talks about the role shared beliefs and interpretations of sexual behaviors play in shaping social interactions of power
	Interactive power: Nonverbal cues	Talks about the presence of nonverbal cues indicating lack of consent.
	Interactive power: Coercion	Talks about a situation of power over the other person in a given context (non-institutional) (e.g., they are in the home of one of them, they are with the group of friends of one of them, age difference…).
	Interactive power: Unwanted sexual consent	Talks about sexual consent being given due to interactions of power even though it is perceived to be unwanted, not freely given
Dialogic Communicative Acts	Dialogic communicative acts: Nonverbal cues	Talks about paying attention to the presence of nonverbal cues indicating consent or lack thereof.
	Dialogic communicative acts: Consent support	Talks about the visibilisation of consent being appreciated.
Ethics	Ethics: Intention	Concerned with perpetrators’ intention to harass or abuse.
	Ethics: Consequences	Concerned with perpetrators being held accountable and/ or taking accountability for the consequences of their actions (responsibility, accountability)

Once the codebook was established, each post was read through several times to, first, determine what main category it fell within and, second, to identify what subcategories were present. In some cases, the same post was identified under more than one category and subcategory.

### 2.3 Quantitative and qualitative analyses

Once all the data was analyzed and codified in dialogue among researchers, quantitative and qualitative analyses were conducted.

The quantitative analysis consisted of two steps. On the one hand, researchers conducted a quantitative analysis to calculate the number of tweets and Instagram posts within each category and subcategory. Second, the engagement of each category and subcategory was calculated, as it is not only relevant to know how many posts have included elements of each of the categories and subcategories, but also what categories and subcategories have received most interactions in each social network. The engagement looked differently in each social network: on Twitter the number of retweets of each tweet was calculated, whereas on Instagram the number of likes and comments of each post was calculated. The following two formulas illustrate how the engagement rate of the posts in each social network was calculated:

Engagement on Twitter


$$\mathrm{Engagement}=\frac{r}{i}\times 100$$

Engagement on Instagram


$$\mathrm{Engagement}=\frac{r+c}{i}\times 100$$

These two formulas were used in their respective social network in two ways: first to calculate the engagement rate of each of the three categories (Power Communicative Acts, Dialogic Communicative Acts and Ethics), and then to calculate the engagement rate of the subcategories within each of the three main categories.

In addition to the quantitative analysis, all posts’ content was qualitatively analyzed in order to identify how they included concepts or examples related to the theory of communicative acts in line with the categories and subcategories established in the dialogic codebook.

### 2.4 Ethical considerations

The extraction of data respected the General Data Protection Regulation (EU) 2016/679 as well as the Terms and Conditions of the selected social networks. Collected data included only public data that users consented to share in each social network. Users’ consent is therefore achieved through their decision to make their posts public. Furthermore, the users’ profiles were not analyzed, and no post, regardless of being public, has been transcribed in this article to ensure users’ anonymity. Ethical approval for this study was obtained through the University of Barcelona.

## 3 Results

As mentioned above, out of the 6,354 Instagram and Twitter posts reviewed, 46 were identified to be concerned with the relevant subject of consent. Of these, 17 have been found on Twitter and 29 on Instagram. Table 3 summarizes the number of tweets and Instagram posts identified as referring to each of the three categories of communicative acts analyzed for this study, as well as the engagement of all tweets and Instagram posts falling within each of the three categories.

**Table 3:** Number and engagement of tweets and Instagram posts.

Twitter			
Category	Number of tweets	Retweets	Engagement (%)
Power communicative acts	15	316	98.1
Dialogic communicative acts	2	4	1.2
Ethics	1	2	0.6
Total	17	322	100

Instagram			
Category	Number of posts	Likes + comments	Engagement (%)
Power communicative acts	27	3,042	
Dialogic communicative acts	0	0	
Ethics	8	1,423	
Total	29	4,602	100

As can be seen in the table, the category in which most posts have been found is Power Communicative Acts for both Twitter and Instagram, with 15 and 27, respectively. Furthermore, the posts classified within this category in both social networks are the ones that have achieved the highest engagement rates, with 98.1 and 66.1%, respectively. These data indicate that, while more posts categorized as Power Communicative Acts have been found on Instagram than on Twitter, within this category the tweets have achieved a higher engagement than the Instagram posts. The next category with the highest number of posts and the highest engagement on Twitter is Dialogic Communicative Acts, with 2 tweets and an engagement rate of 1.2%, also the third highest one in this social network. However, the second highest category on Instagram is Ethics, with 8 posts and 30.9% engagement rate, also the second highest one. Last, the category with the least number of tweets is Ethics, with only one tweet and an engagement of 0.62%. On the other hand, the category with the lowest number of posts on Instagram is Dialogic Communicative Acts, with no posts identified as such. In this section we break down these data into the specific subcategories and include examples of the content of the posts categorized within them.

### 3.1 Power communicative acts

Within Power Communicative Acts there is a similar tendency in both social networks, with Interactive Power being higher than Institutional power: there are 12 tweets with 96.2% engagement and 27 Instagram posts with 66.1% engagement categorized as Interactive Power, as opposed to 3 tweets with 1.9% engagement and 4 Instagram posts and 3% engagement categorized as Institutional Power. In both social networks it can be seen that the engagement of the tweets and Instagram posts including elements of Interactive Power is much higher than the engagement of the tweets and Instagram posts referring to Institutional Power.

Taking a closer look at the subcategories within Interactive Power, the tendency varies across the two social networks. On Twitter, most posts refer to *sexual scripts* (9), but the engagement of the posts referring to *sexual scripts* is the lowest one, with 16.8% engagement rate. In turn, the tweets with the highest engagement rate are subcategorized as *coercion*, with 97% engagement and 5 tweets. In between both subcategories we find tweets referring to *nonverbal cues*, with 2 tweets and 82.9% engagement. No tweets were subcategorized as *unwanted sexual coercion*. On Instagram, however, the subcategory *sexual scripts* is the one with most posts (23) and the highest engagement rate (72.1%). The subcategory with the second highest engagement rate is *unwanted sexual consent* (27%), although only 3 posts referred to it. Next, we find posts referring to *coercion*, with 5 posts and 18.5% engagement rate, and last we find posts including elements of *nonverbal cues*, with 4 posts and 18% engagement.

Among the detailed qualitative analysis of the posts that engage with concepts linked to Interactive Power, some of them spoke about the hindrance that insisting, coercing, manipulating or otherwise pushing victim-survivors survivors into doing something poses to consent. A few posts also spoke of the lack of consciousness of the victim-survivor survivor either due to alcohol, drugs or being withheld information from as a barrier to consent. Most posts within this subcategory spoke about the problems that arise when we look at communicative acts of the victim-survivor survivor—their choice of clothing was a common recurring example -, even blaming them as supposedly providing consent, rather than the Interactive Power used by the perpetrator. On the one hand, most of these posts condemned that warning potential victim-survivors survivors of the common excuses perpetrators use for their misconduct perpetuates the notion that the victim-survivor survivor must be responsible for things that are out of their control and/or restricts their freedom. On the other hand, they argued that this narrative is completely ineffective in preventing abuse and that perpetrators who are willing to use Interactive Power will do so regardless of whatever the victim-survivor survivor chooses to do.

A few posts protested counterproductive narratives that blame victim-survivors survivors for not expressing their refusal of consent clearly enough. They pointed out that the perpetrators’ willingness to use Interactive Power is what makes it hard or impossible for the victim-survivor survivor to express a clear no. Notably, some posts rejected the proposal of an app supposed to register consent. These posts pointed out that people may be coerced into registering their consent on this app even when they do not wish to do so, and that even when registering their consent willingly at first, they may change their mind during the encounter. Thus, the app seems to erase all the non-verbal cues and possible situations of Interactive Power that hinder consent and turn it into a legal trap.

Among the posts that pointed out the issue of Institutional Power, varying examples can be found, from recurring sexual abuse suffered by incarcerated women, to sexual harassment at work, over domestic violence. For instance, one tweet shared an article outlining in detail how an NYU professor sexually harassed one of her advisee students repeatedly. This exemplifies how the power asymmetry, with the professor having significant control over the student’s career, made it difficult for the student to escape the situation. Many of the tweets within this category question whether the power asymmetries provide victim-survivors with the capacity to say “no.”

Others were oriented toward helping social media users identify different elements, such as power imbalances created by hierarchical structures that can promote coercion and therefore make consent impossible. In addition, some of the posts referring to Institutional Power also contained the concept of Interactive Power. The same post about the professor, for instance, also fell into the category of Interactive Power through insisting, coercing, manipulating or other verbal and non-verbal aggression. The case is a good example given that the professor’s persistence combined with her position of power made her manipulative abilities that much more effective in getting her way.

### 3.2 Dialogic communicative acts

No Instagram posts were identified as referring to Dialogic Communicative acts. As for Twitter, 1 tweet was categorized as *nonverbal cues*, and 1 as *consent support*. However, the difference in terms of engagement between both tweets is quite high: the tweet categorized as *nonverbal cues* received a 25% engagement rate, whereas the one categorized as *consent support* received a 75% engagement rate.

In terms of the content, the tweet categorized as *nonverbal cues* refers to the need to pay attention to elements such as signs of distress or discomfort to identify whether the other person is giving their consent or not. The tweet categorized as *consent support*, in turn, refers to the importance of taking consent into account in conversations about relationships, highlighting consent as something positive and necessary.

### 3.3 Ethics

Last, the subcategory within Ethics that has achieved the highest number of posts and of engagement is *consequences*. In fact, on Twitter the only tweet referring to Ethics has been identified within this subcategory, hence having 100% of the engagement. On Instagram, there are six posts referring to *consequences*, having reached an engagement rate of 73%, and only two posts have been subcategorized as *intentions*, having achieved 27% engagement.

Most posts within this category spoke about the need for potential predators to take responsibility or be held accountable by others for the outcome of their actions. A few posts emphasized that the intention of abusing, harassing or being otherwise sexually violent is wrong. In turn, most of the posts that engaged with the concept of Ethics did so to emphasize the need for an ethics of consequence to be applied to our understanding of consent. Most examples in this category highlight the importance of holding perpetrators, rather than the victim-survivor, accountable. For instance, many of the posts referred to victim-survivors’ communicative acts which are often used as excuses signaling consent advocated for shifting the blame from the victim-survivor to the perpetrator, holding the latter accountable for ensuring that a clear consent is provided.

## 4 Discussion

This study makes a previously underexplored contribution to the sociological research literature on how citizens interact about sexual consent in social media by analyzing to what extent and how the posts analyzed use characteristics and concepts of the theory of communicative acts. The study found that, among all the tweets and Instagram posts reviewed and analyzed, 46 referred to some concepts related to the theory of communicative acts.

Despite the low number of posts that contain key concepts and elements of the theory of communicative acts as compared to all the data extracted and analyzed, these findings provide important advancements to the literature on sexual consent. For many decades, the perspective on speech acts has been predominant in the understanding and conceptualization of sexual consent, that is, focusing only on victims’ words (Curtis and Burnett, 2017; Flecha et al., 2020). Yet the posts analyzed indicate that, while still a small number of citizens, there are already a few social media users who claim for the need to account for much more than words and, most importantly, who are already identifying other elements of communication as essential to identify and understand consent (Soler and Flecha, 2010). While it remains unknown to what extent they know the theory behind the concepts and elements those social media users bring into social media interactions, the findings show that they do use such elements in relevant everyday interactions in social networks, which are themselves a relevant site to better analyze how citizens conceptualize and understand sexual consent (Aurrekoetxea-Casaus, 2020; Kettrey et al., 2021; Worthington, 2020).

Most posts engaged with concepts and elements of Power Communicative Acts (Flecha et al., 2020). In particular, the high amount of references to the concept of Interactive Power shows that its use in sexual encounters lies at the center of the issue among participants. Whether it is through deeply embedded narratives that allow covert manipulation or through more obvious displays of malice, the mere presence of Interactive Power shows that consent is compromised. Moreover, the recurring themes in the posts reflected a general concern with victim-survivor-blaming attitudes and demanded the attention be shifted back onto the perpetrators and their use of Interactive Power. Many complained that women’s state of dress, undress or physical appearance in general—e.g. being curvy—was commonly presented as the central reason for their encounter with violence. These posts sought to emphasize that even when covering up and dressing in what is perceived as a sexually unappealing way sexual violence persists, and that this logic is therefore flawed. Furthermore, they argued that regardless of what victim-survivors wear, the use of Interactive Power by the perpetrator is exclusively at fault for the violence. This recurring argument rejects the concept of unintentional illocutions (Cowart, 2004) and instead denounces the use of manipulation, coercion, insistence or any other verbal or non-verbal types of aggression as well as lying, deceiving or taking advantage of an unconscious victim-survivor.

The line of reasoning of the posts speaking about Interactive Power aligns with existing research that suggests that perpetrators are capable of understanding refusal of consent both when it is explicit and implicit in complex or non-verbal forms of communication. Thus, they not only choose to ignore the refusal within these ambivalences but moreover use them to hide their action behind the excuse of misunderstanding (Harris, 2018). Furthermore, by rejecting analyses centered on the expression of consent and/or requesting to shift attention toward the perpetrators, most of the posts analyzed align with the literature that places Interactive Power as a crucial motor to the establishment of a Power Communicative Act (Flecha et al., 2020).

The concept of Institutional Power, also present in the data analyzed, was of much concern during the #metoo movement. It became especially mediatic through cases of sexual violence such as those perpetrated by the recently incarcerated producer Harvey Weinstein, who used his institutional power to abuse, harass and rape several members of the Hollywood industry (Overbey, 2022). The hierarchies that exist within institutions can restrict the freedom of an employee, for instance, in rejecting sexual advances and clearly expressing their discomfort and consent refusal. The tweet that shared an article outlining the case of professor-advisee harassment at NYU was precisely concerned with this.

On the other hand, a few posts spoke about the need for more Dialogic Communicative Acts that enable the establishment of consent to sexual encounters. While some of these posts also promoted affirmative consent, the emphasis was on asking for consent, or ensuring one obtained consent before continuing with the sexual encounter rather than on the clear and verbal expression of consent or consent refusal. Along these lines, only considering affirmative consent has previously shown barriers that have been evidenced also in the present study (Willis and Jozkowski, 2018). The legislation concerning affirmative consent constituted a notable advance on previous law (North, 2022), but nonetheless, it remains insufficient. In addressing concrete actions, the need to clarify consent (Curtis and Burnett, 2017) with considerations that go beyond affirmative consent and to broaden its conception to include the factors that influence the affirmation itself has been highlighted. Further studies may consider comparing the engagement of affirmative consent with broader Dialogic Communicative Acts that seek mutual understanding, read verbal and non-verbal cues as well as context.

Last, some posts engaged with the concept of Ethics regarding consent. While many limited their focus to pointing out how wrong it is to ignore consent, the majority of posts that spoke about Ethics went further. They emphasized an ethics of consequences (Weber, 1930) and pointed out that perpetrators must be held accountable and take responsibility for their actions. One could argue that the fact that the very act of taking responsibility for ensuring consent is genuine is what differentiates a Power Communicative Act from a Dialogic Communicative Act. After all, allowing one’s position of power to compromise their counterpart’s freedom to decide or, in turn, using Interactive Power is a choice made by the perpetrator. Somebody who does not wish to traumatize their counterpart will take responsibility for the consequences of their actions if they make a mistake (Flecha et al., 2020).

These findings hold implications for different disciplines and areas related to sociology, especially for education. Much research has claimed the need for sex education to delve deeper on issues related to consent, as many adolescents and youth still feel they have nuanced or incomplete understandings of consent (Richmond and Peterson, 2020). Research has also pointed out how challenging teaching about consent is (Curtis and Burnett, 2017; Healy Cullen et al., 2023). The findings in this study shed some light on diverse social media users’ complex and fine-tuned understanding of sexual consent which takes up elements of the communicative acts theory, which has already shown to be pivotal in providing a more rigorous analysis and conceptualization of consent than those based only on words (Flecha et al., 2020; Soler and Flecha, 2010). Further research should continue along this line to delve deeper on how more and more adolescents and youth can be trained based on scientific evidence to introduce such elements into their understanding and identification of consent and, along this line, the social impact that introducing them has on their own sexual-affective relationships.

### 4.1 Limitations

Some limitations and prospective research need to be considered. The number of posts analyzed for this study is relatively low, partly since the data was selected from a separate study. While the topic of the other study substantially overlapped with this one, the content of the extraction was not tailored to the focus of this study. Furthermore, the dates of the extraction will reflect the sentiment of that particular moment. Thus, for more conclusive comparative results, further extractions aimed at the study of consent and sexual violence, as well as an extraction over a more prolonged period of time will make for a more representative study. This study has used a range of types of Interactive Power that are of concern to the social media users represented in this study. A wider range of examples of consent and consent refusal can deepen our understanding of the dynamics of Power Communicative Acts through Institutional and Interactive Power as well as Dialogic Communicative Acts. A comparative study that observes the difference between posts concerned with sexual violence generally and those talking about consent specifically may produce more answers. Further studies could also review which mechanisms best ensure the establishment and normalization of Dialogic Communicative Acts within institutions. Finally, prospect studies may also consider reviewing how many of the posts that propose a solution do so either by suggesting affirmative consent exclusively, or affirmative consent as part of a Dialogic Communicative Act.

## 5 Conclusion

Scientific contributions from different disciplines, particularly sociology, regarding consent have come a long way. On the one hand, this article has briefly reviewed the developments in linguistics, and specifically pragmatics and sociolinguistics, which successfully describe the complexities of the communication of consent. However, it has showed the essential contributions from sociology to better understand and conceptualize those complexities by engaging with the literature that has developed an effective concept of Communicative Acts, which offers an accurate differentiation between egalitarian and violent sexual encounters, regardless of how the victim-survivor expressed consent or consent refusal. As a step forward, this study informs how social network debates fit the Communicative Acts analysis and can thus be conducive to improving popular understanding and respect for consent. In all, the SMA shows that the concept of Communicative Acts—which includes the importance of the ethics of consequence as well as the concepts of Power and Dialogic Communicative Acts—resonates largely with popular conversations about freely given consent that is based on egalitarian relationships among the posts analyzed.

## Data Availability

Publicly available datasets were analyzed in this study. The data that support the findings of this study are openly available in Zenodo at: https://zenodo.org/record/4729725#.Yh14HBPMJOc, doi: 10.5281/zenodo.4729725.
